# First person – Omkara Lakshmi Veeranki

**DOI:** 10.1242/dmm.043521

**Published:** 2019-12-17

## Abstract

First Person is a series of interviews with the first authors of a selection of papers published in Disease Models & Mechanisms, helping early-career researchers promote themselves alongside their papers. Omkara Lakshmi Veeranki is first author on ‘ A novel patient-derived orthotopic xenograft model of esophageal adenocarcinoma provides a platform for translational discoveries’, published in DMM. Omkara Lakshmi is a postdoctoral fellow in the lab of Dr Dipen Maru at The University of Texas MD Anderson Cancer Center, Houston, TX, USA. The focus of her research is understanding key molecular targets and the alterations in gene expression that contribute to pathogenesis of gastrointestinal cancers, which could be used for developing anticancer therapeutics.


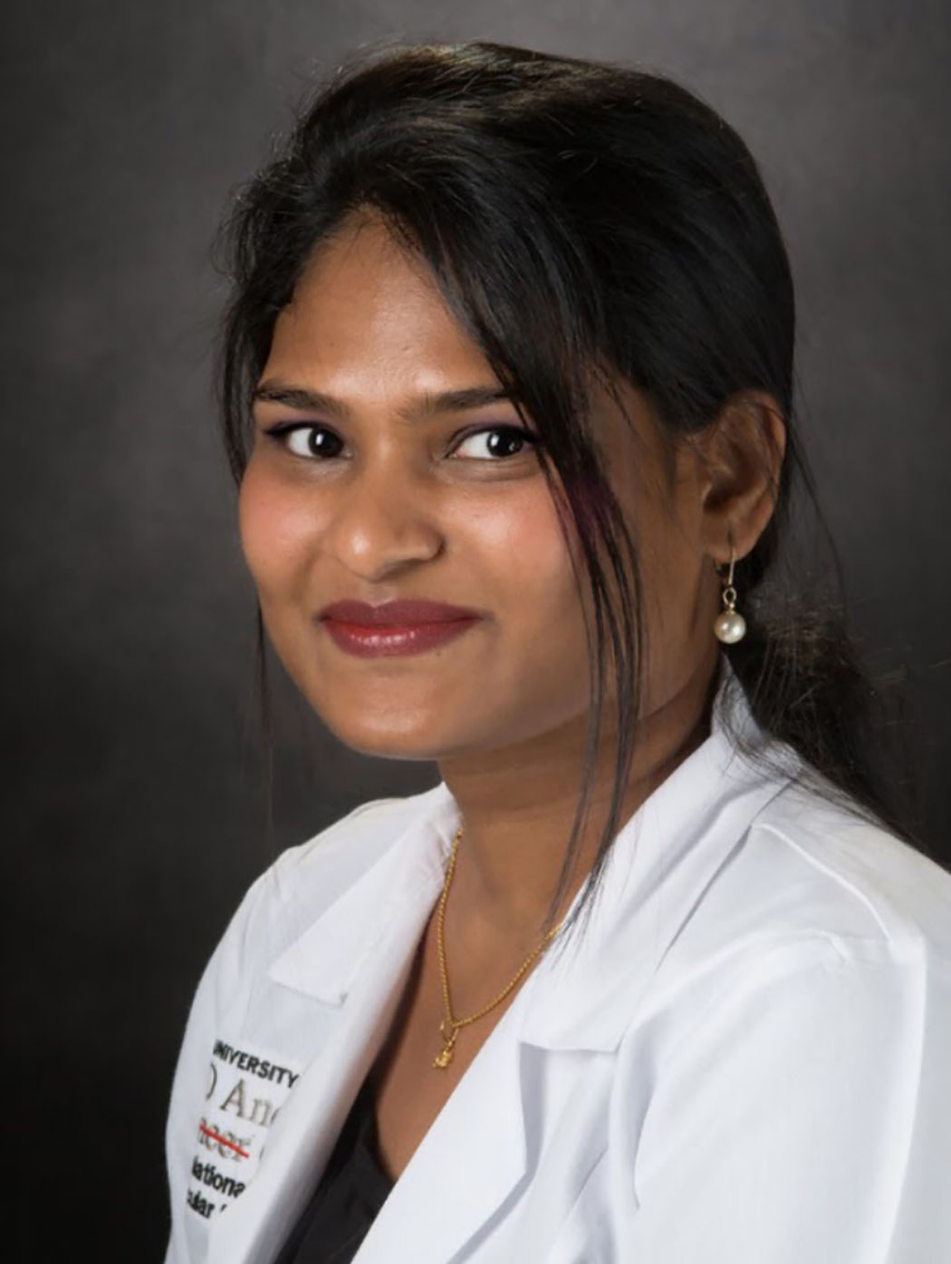


**Omkara Lakshmi Veeranki**

**How would you explain the main findings of your paper to non-scientific family and friends?**

Nearly 1.6 million new cases and more than 1 million deaths related to gastroesophageal junction (GEJ) cancer are estimated for 2019. Despite treatment advances, GEJ cancer remains a global health concern. Limited progress in treatment options is due to molecular complexity of this cancer and a lack of models that fully mimic human disease. Current GEJ models have limited applications in tumor microenvironment (TME), immune oncology and metastatic studies. Thus, we established the patient-derived orthotopic xenograft model (PDOX) for GEJ cancer. Patient tumors engrafted at the GEJ in immunodeficient mice generate a mouse model that is close to the genetic profile of the patient tumor and serves as a great tool to further our understanding of the molecular basis of GEJ cancers, which can be exploited for developing new therapies. We found that the GEJ PDOX model mimicked the tumor growth pattern and recapitulated the hallmarks of an aggressive GEJ cancer as seen in the TME of the patient tumor. Next-generation sequencing and immunohistochemical analysis indicated that the GEJ PDOXs were similar to patient tumors at the molecular and histological level. Targeted radiotherapy designed to be similar to clinical application in the GEJ PDOX model demonstrated partial response to treatment without any radiotoxicities in the adjoining soft tissues. The pursuit for developing novel mouse models has invigorated the field with newer transgenic mice models, and there has been a rise in publications with patient-derived xenografts. In our study, we show that the PDOX model of GEJ and esophageal adenocarcinoma exhibits remarkable fidelity to human disease, providing a novel tool for translating bench findings.

**What are the potential implications of these results for your field of research?**

Cancer is associated with a large socioeconomic burden. Genetic profiling of patient tumors has provided unprecedented capacity to characterize tumors at a molecular level, yet the ability to translate these findings in a clinical setting is limited. To enable a cost-effective real-time translation of personalized therapeutic approaches to patient care will require the integration of mouse avatars that very closely resemble patient disease, such as the PDOX model we established.

Further, with the development of emerging targeted therapies and multimodal treatment approaches, the need is growing for preclinical models that accurately represent the molecular subtype of the disease to allow correlation with treatment sensitivity and patient clinical history. We showed the maintenance of the radiological, pathological and tumor growth characteristics, even with the highly heterogeneous GEJ cancers, emphasizing the use of PDOX models as a powerful investigational platform that may be used to evaluate new therapies.

**What are the main advantages and drawbacks of the model system you have used as it relates to the disease you are investigating?**

The main advantage of this model system is the practical applications in developing novel approaches in the management of GEJ adenocarcinoma. This model can be used to study biology of the disease, multi-modal targeted therapies, intratumoral oncolytic viruses, drug screenings, clonal evolution, neoangiogenesis and the microenvironment, and for running co-clinical trials mirroring human trials. The drawbacks of the current model are that it lacks an active immune system, thus impeding immunotherapy studies and projects focused on the crosstalk between immune cells and tumors. Therefore, our future studies are directed towards optimizing this model in a humanized mouse that will address these shortcomings. One limitation in our study is that this model did not demonstrate distant metastasis. However, metastasis developing from a PDOX model like this one would be more representative of a stage-IV esophageal adenocarcinoma than any other available mouse models.

“[…] the utility of this model in creating mini-avatars or mini-clones to test therapeutics that have high translational value was my ‘aha’ moment.”

**What has surprised you the most while conducting your research?**

Radiotherapy is an important treatment modality. But the radiotoxicities and tumors developing radioresistance have to be taken into consideration to discover molecular targets of radiosensitization. Most mouse models for GEJ adenocarcinoma harbor subcutaneous tumors that don't represent the architecture and the locoregional environment of the tumors arising in patients. Thus, these subcutaneous models pose significant limitations in predicting radioresponse in the clinic. I was surprised to see response to targeted radiotherapy by a single fraction of 10 Gy and re-growth of tumors at a later time point in our PDOX system, suggesting that one dose of 10 Gy is sufficient to shrink tumors but not enough to completely ablate the tumor. This finding is very similar to the observations in clinics, where patients receive radiotherapies involving several fractions. In addition, I was fascinated to the see the remarkable resemblance of this mouse model to the human disease and how this GEJ PDOX could preserve the molecular patterns present in donor tumors. This high degree of resemblance is promising, especially because GEJ cancers are highly heterogeneous and no one treatment would work for all GEJ adenocarcinoma patients. Thus, the utility of this model in creating mini-avatars or mini-clones to test therapeutics that have high translational value was my ‘aha’ moment.

**Figure:**
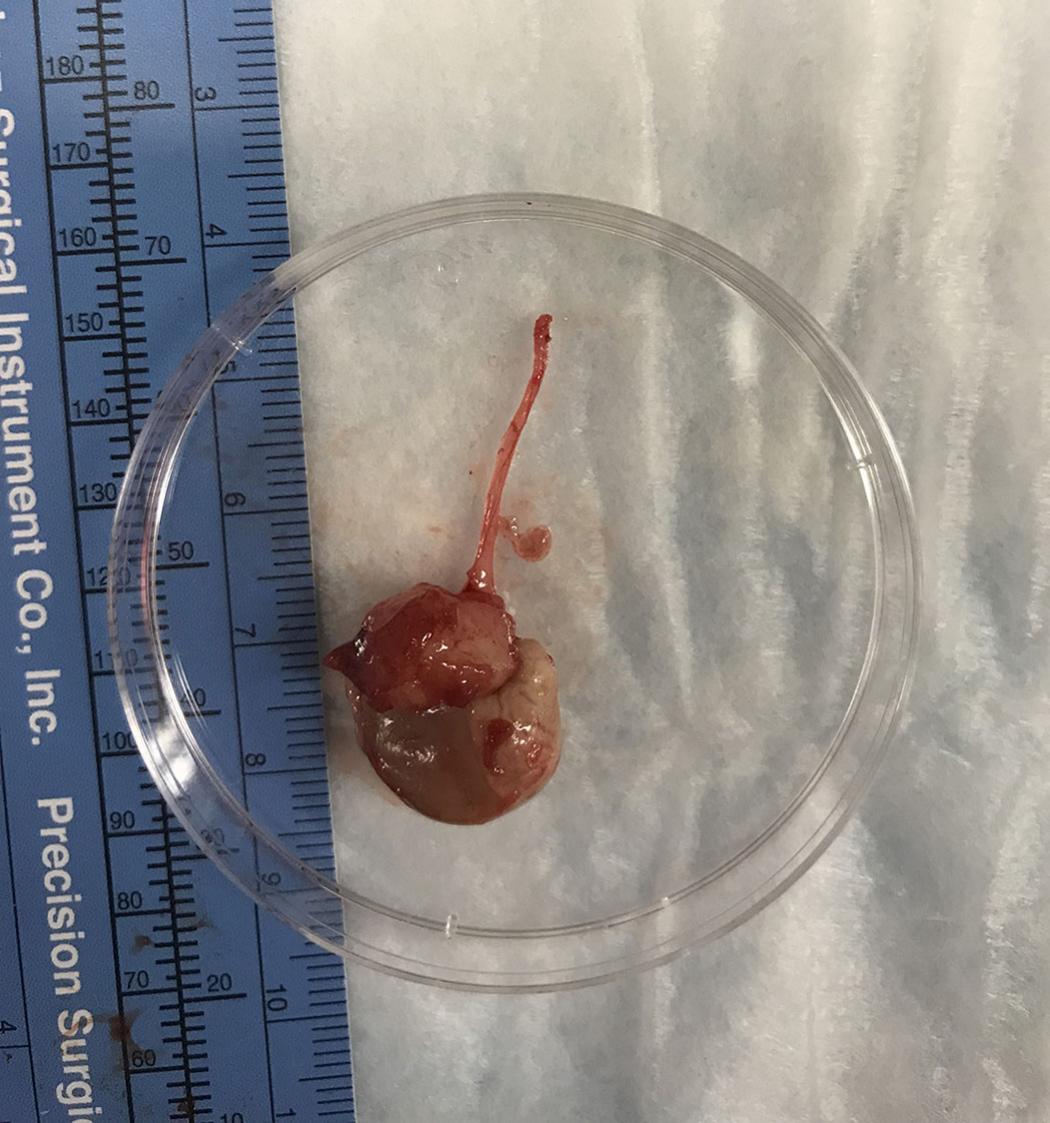
**Mouse avatar of gastroesophageal junction cancer.**

**Describe what you think is the most significant challenge impacting your research at this time and how will this be addressed over the next 10 years?**

The role of the immune system and the mechanisms that elicit an immune response to various therapies hasn't been fully studied, primarily due to lack of an effective genetically engineered mouse model for GEJ adenocarcinoma. A deep understanding of the dynamic immune landscape and immune cell interactions with the stromal and tumor cells will advance our knowledge significantly in designing immunotherapies for GEJ cancer. With the help of a humanized mouse model paired with modern flow-cytometric analyses and sequencing tools, I think that we will have a comprehensive understanding of how this cancer escapes being identified by the immune system. This will eventually help in developing immune modulators for GEJ cancers.

“[…] a network of mentors and sponsors outside of our immediate environment can enhance the experience of early-career scientists in identifying great opportunities and achieving success in scientific ventures.”

**What changes do you think could improve the professional lives of early-career scientists?**

I am thankful to DMM for promoting early-career researchers alongside their publications. My institute and my department in particular encourage young scientists by providing an ecosystem that is multidisciplinary. My mentor, Dr Dipen Maru, has been instrumental in creating opportunities that enhance my scientific and leadership enterprises in furthering my research pursuits. I believe that a network of mentors and sponsors outside of our immediate environment can enhance the experience of early-career scientists in identifying great opportunities and achieving success in scientific ventures. An organized mentor-mentee program that matches the pair from different institutes (academic and industry) will help propel young scientific talent. Also, a discipline-wide initiative in inviting first author (early-career scientists) presenters as invited speakers along with their mentors helps to put the early-career scientists in the forefront, promoting them and their work.

**What's next for you?**

The current PDOX model of GEJ cancer was developed using immunocompromised mice that lack a functional immune system. In order to study immunotherapies and the mechanisms by which immune modulators mimic immune response in TME, the next thing would be to develop the GEJ PDOX model in a humanized mouse that will be able to mount an anticancer immune response. A humanized GEJ PDOX mouse model will comprise of infiltrating human immune cell subsets, chemokine signaling, lymphangiogenesis and tumor-associated fibroblasts, mimicking a more realistic TME. In addition, now that the PDOX model of GEJ cancer is established, we will continue with high-throughput drug screening using PDOX models harboring diverse genetic mutations to demonstrate reproducibility and patient translatability in identifying drug responses associated with specific mutations. Personally, my overarching goal is to make significant contributions in the field of comparative medicine by developing new therapeutic strategies tailored specifically for the cancer patient's genetic makeup to ultimately improve patient outcome. The next goal is to transition into an independent investigator.
